# Viral infection and thyroid disorders: a narrative review

**DOI:** 10.3389/fmicb.2025.1625179

**Published:** 2025-06-13

**Authors:** Yitian Li, Weiyang Li

**Affiliations:** Department of Hygiene, Public Health College, Jining Medical University, Jining, China

**Keywords:** viral infection, autoimmune disease, thyroid disease, thyroid cancer, anaplastic transformation

## Abstract

Thyroid disorders, including thyroid dysfunction, autoimmune thyroid diseases (AITDs), and thyroid cancers (TCs), are receiving increasing attention as significant public health concerns. Viral infection can interfere with normal immune function by activating innate and adaptive immunity, causing endocrine disorders. As an important endocrine gland, thyroid function is easily affected by viral infection, inducing the formation of autoantigens by mimicking thyroid components, thereby promoting the development of AITDs. Viruses like herpes simplex virus (HSV), Epstein–Barr virus (EBV), Hepatitis C virus (HCV) and human parvovirus B19 (B19V) are potential candidates associated with AITDs. These viral infections also play a key role in tumor pathogenesis, where chronic infection or reactivation of viruses can change the immune microenvironment in the body and promote the occurrence and development of cancer. Numerous studies have confirmed the associations between various viruses, such as B19V, EBV, HSV, and HCV, with TCs. This review examines the impacts of viral infections on thyroid function and the underlying mechanisms involved, and also analyzes the common characteristics and mechanisms of viral infection-induced TCs. By analyzing the existing research hypotheses, we summarized the possible mechanisms of viral infection-induced thyroid disorders and also identified the potential role of viral infection in the process of anaplastic transformation of TC. This information provides insights into the model of multistage carcinogenesis of TCs, especially the mechanism of the transition from differentiated to undifferentiated or anaplastic TCs.

## 1 Introduction

Thyroid disorders, such as thyroid dysfunction, autoimmune thyroid diseases (AITD), and thyroid cancers (TCs), are increasingly becoming a public health problem with AITD affecting approximately 5% of the population, and TCs accounting for up to 3% of the overall cancer burden. In 2020, the age-standardized incidence rates of TC were 3.1 per 100,000 men and 10.1 per 100,000 women (Pizzato et al., 2022). Although overdiagnosis may partially explain the increased global incidence of TC by 20% from 1990 to 2013 (Kim et al., 2020), most medical practitioners agree that there is a genuine rise in the prevalence of thyroid-related pathologies. Autoimmune thyroid diseases include hyperthyroidism Graves’ disease (GD), and hypothyroidism Hashimoto thyroiditis (HT). The GD is triggered by thyrotropin receptor antibodies (TRAbs) that target the thyroid-stimulating hormone receptor (TSHR), which is responsible for the major pathogenesis of GD (Furmaniak et al., 2015). On the other hand, HT pathologically consists of TH1 lymphocyte-mediated infiltration of the thyroid gland and autoantibodies against thyroid peroxidase and thyroglobulin, which are the key proteins involved in thyroid hormone synthesis (Walsh et al., 2010).

The TCs are classified into three distinct categories, including benign, low-risk and malignant neoplasms (Baloch et al., 2022). Among the malignant neoplasms, papillary thyroid carcinoma (PTC) accounts for 85% of the TCs, follicular thyroid carcinoma (FTC) contributes 10% of the TCs, while other cancer types (OCA) are responsible for 5% of the TCs. Most TCs exhibit a 98% favorable prognosis, with a 5-year survival rate (Ludgate et al., 2024), except high-grade and poorly differentiated TCs (Akaishi et al., 2019), which have high proliferation rates, necrosis and specific growth patterns. Anaplastic thyroid carcinoma (ATC), which is a rare undifferentiated subtype, accounts for 1% of all TCs and is associated with a poor prognosis. Research has shown that the extracellular signal-regulated kinase–kinase-mitogen-activated protein kinase (ERK–MAPK) and phosphatidylinositol 3-kinase (PI3K) pathways are the key drivers of malignant transformation in TC (Cancer Genome Atlas Research Network, 2014). The differentiated TC can be further classified into BRAF-like tumors, predominantly comprising PTC, and rat sarcoma (RAS)-like tumors, which include follicular variants of PTC and tumors exhibiting a follicular growth pattern (Fagin et al., 2023). Other genetic alterations, including mutations in the telomerase reverse transcriptase gene (TERT) promoter and tumor protein 53 (TP53) mutations, may also contribute to disease progression, which are related to patient survival, dedifferentiation and anaplastic transformation (Melo et al., 2014; Kunstman et al., 2015). The clinical features of all thyroid disorders, including thyroid dysfunction, AITD, and common TCs, have been summarized in Table 1.

**Table 1:** Features of thyroid disorders discussed in this review.^a^

Name	Features
Subclinical hypothyroidism	Normal free tetraiodothyronine (fT4) ± free triiodothyronine (fT3) levels and TSH↑
Overt hypothyroidism	Low fT4 ± fT3 levels and the compensatory TSH↑
Subclinical hyperthyroidism	Normal fT4 ± fT3 levels and TSH↓
Overt hyperthyroidism	Increased fT4 ± fT3 levels and the compensatory TSH↑
Subacute thyroiditis	It occurs concurrently or within a few days of viral infection. Symptoms include neck pain, fever, and manifestations of thyrotoxicosis
Painless thyroiditis	It occurs within the context of severe SARS-CoV-2 infection. Symptoms include the absence of neck pain and presentation of postpartum thyroiditis
GD	Hyperthyroidism can be caused by the presence of TRAb, which binds to the TSHR and mimics the action of TSH
HT	Autoimmune hypothyroidism arises from the T helper 1 cell-mediated autoimmune impairment of the thyroid gland through mechanisms involving apoptosis and necrosis. The detection of autoantibodies against thyroid peroxidase and thyroglobulin may serve as indicators of a pre-disease state
PTC	Follicular cell-derived TC, which accounts for 85% of all TCs, is primarily caused by the activation of the ERK/MAPK signaling pathway. This activation occurs through RAS and BRAF mutations as well as gene fusions involving NTRK and RET
FTC	Follicular cell-derived TC, accounting for 10% of all TCs, is primarily attributed to the activation of the ERK/MAPK signaling pathway resulting from the mutations and gene fusions of RAS involving PAX8–PPARγ rearrangements
ATC	A rare (less than 1%) and highly aggressive form of TC that harbors concurrent TP53 and TERT promoter mutations, along with gene mutations activating the PI3K–AKT and ERK/MAPK pathways

a The clinical features of thyroid disorders in the table are mainly cited from two review articles (Ludgate et al., 2024; Lui et al., 2024). fT4, free tetraiodothyronine; fT3, free triiodothyronine; TSH, thyroid-stimulating hormone; TSHR, thyrotropin receptor; GD, Graves’ disease; HT, Hashimoto’s thyroiditis; PTC, papillary thyroid cancer; FTC, follicular thyroid cancer; ATC, anaplastic thyroid cancer; ERK, extracellular regulated protein kinases; MAPK, mitogen-activated protein kinase; BRAF, serine/threonine-protein kinase B-Raf; RAS, rat sarcoma; RET, rearranged during transfection; NTRK, neurotrophins receptor kinase; PAX8, paired box 8; PPARγ, peroxisome proliferators-activated receptorγ; TERT, telomerase reverse tranase; PI3K, phosphatidylinositol 3-kinase; AKT, protein kinase B.

Viruses can trigger thyroid disorders in various ways, including activation of the innate and adaptive immune responses, leading to AITD. Viruses like Herpes simplex virus (HSV), human T-cell lymphotropic virus-1 (HTLV-1), Epstein–Barr virus (EBV), rubella, mumps virus, and enteroviruses have been implicated in HT (Desailloud and Hober, 2009). Retroviruses like HTLV-1, Simian virus 40 (SV40), and human immunodeficiency virus (HIV) are related to GD (Desailloud and Hober, 2009), while Hepatitis C virus (HCV) and human parvovirus B19 (B19V) are potential candidates for AITD (Morohoshi et al., 2011). Additionally, acquired immunodeficiency syndrome (AIDS) and HIV infections have been linked to various thyroid disorders (Parsa and Bhangoo, 2013). Viral infections also play a key role in tumor pathogenesis. For instance, EBV is associated with Burkitt lymphoma (Almeida et al., 2017), while BK virus (Pierangeli et al., 2015), B19V (Mostafaei et al., 2020), EBV (Antonelli et al., 2007; Etemadi et al., 2017), HSV (Read and Douglas, 2014), and HCV (Kitsou et al., 2021) are associated with TC. Furthermore, chronic viral infections also account for 20–25% of all human cancers (Moghoofei et al., 2019). These viruses exert their influence on the development and progression of TC through one or more of the molecular alterations. For example, EBV affects TC by increasing Epstein–Barr nuclear antigens to modulate BRAF activity, and inducing the expression of latent membrane proteins to activate the NF-κB signaling pathway (Homayouni et al., 2017; Almeida et al., 2019).

With the advancement of knowledge regarding the virus and its interaction with the immune system, a theory has emerged concerning the relationship between certain viral infections and thyroid disorders. In this review, we examined the impacts of viral infections on thyroid function and the underlying mechanisms involved. The references were primarily sourced from online databases, including Google Scholar, Web of Science, ScienceDirect, and PubMed. The search was limited to studies published in English or Chinese and those conducted on human subjects. Only articles and reviews were selected for analysis, while symposium or conference papers were excluded from consideration. The review also explored the diagnostic and clinical implications of viral infection in thyroid diseases by analyzing the association between viral infection and thyroid disorders.

### 1.1 Epstein–Barr virus

Epstein–Barr virus is a B-lymphotropic virus and a common human herpesvirus that infects most of the global population during their lifetime, thereby establishing a lifelong latent infection following an acute phase. The virus, which was initially identified *in vitro* in cells from Burkitt’s lymphoma tissue through electron microscopy (Lu et al., 2024), enters the B cells through the interaction of its envelope glycoprotein gp350 with the CD21 receptor present on the cell surface. Though the virus is typically transmitted through exposure to oral secretions, with the oropharyngeal B cells as the primary site of infection in humans (Cohen, 2000), its infection is involved in several major autoimmune diseases, such as multiple sclerosis (MS), systemic lupus erythematosus, and rheumatoid arthritis (Sorgato et al., 2020). The virus infection may also contribute to both the initiation of autoimmunity and the exacerbation of disease progression.

Epstein–Barr viral infection is associated with the pathogenesis of many thyroid diseases, including HT, thyroid adenomas, and thyroid carcinomas, suggesting a potential role in the progression of these lesions (Almeida et al., 2020; Karaarslan et al., 2024; Lu et al., 2024). Mertowska et al. have hypothesized that EBV increases the expression of intracellular Toll-like receptors (TLRs), thereby contributing to the development of HT (Mertowska et al., 2024). Their cohort study involving 74 volunteers suggest that the reactivation of EBV is associated with a higher percentage of T helper, cytotoxic T, and B lymphocytes expressing TLR3, TLR7, TLR8, and TLR9 in HT, potentially aggravating the autoimmune response. Therefore, the activity of intracellular TLRs and reactivation of EBV may play a potential role in the pathogenesis and progression of HT (Table 2).

**Table 2:** The key functions of viral infections and their relationship to thyroid diseases.

Virus infection	Direct cell entry	Cytokine modulation	Thyroid autoantibody	Thyroid dysfunction	AITD	TC	References
Epstein–Barr virus (EBV)	√	×	√	√	√	√	Nagata et al. (2015), Mertowska et al. (2024), Almeida et al. (2020), Karaarslan et al. (2024), Lu et al. (2024)
α-human herpesvirus (αHHV)	√	√	√	√	√	×	Smith et al. (2000), Sanfilippo et al. (2004), Thomas et al. (2008), Jensen et al. (2010)
HHV-6A	√	√	×	√	√	√	Caselli et al. (2012), Jasirwan et al. (2014), Eliassen et al. (2018), Mardente et al. (2023)
Human papillomavirus (HPV)	√	×	×	√	√	×	Archin Dialameh et al. (2021), Stamatiou et al. (2015), Hviid et al. (2018)
Hepatitis C virus (HCV)	×	√	√	√	√	×	Antonelli et al. (2004), Antonelli et al. (2007), Jadali (2013), Ferrari et al. (2019)
Human immunodeficiency virus (HIV)	×	×	×	√	×	×	Gangannavar et al. (2020), Dutta and Kalita (2023), Mohamed et al. (2025)
Human parvovirus B19 (B19V)	√	√	√	√	√	×	Wang et al. (2010), Weider et al. (2022), Chandramoorthy et al. (2024), Tzang et al. (2025)
SARS-CoV-2	√	√	×	√	√	×	Brancatella et al. (2020, 2023), Soldevila et al. (2022), Clarke et al. (2022), Lui et al. (2024)

The EBV may also trigger the pathogenesis of GD through its reactivation, which produces thyrotropin receptor autoantibodies (TRAbs), leading to GD (Nagata et al., 2023), and via its infection that stimulates B cells to produce TRAbs in GD (Nagata et al., 2015). Though both TRAb production and EBV reactivation occur simultaneously, the EBV reactivation can induce the release of TRAb in both TRAb (+) and EBV (+) cells from GD patients (Nagata et al., 2015), indicating that the immune response caused by this virus reactivation may play a crucial role in the GD pathogenesis.

The EBV infection induces molecular and morphological changes in TC cells, and also alters the expression of oncogenes. For instance, EBV-infection caused syncytium formation and pyknotic cell shrinkage in TPC-1 and 8505C cells and also altered the expression of TP53 and neuroblastoma RAS viral oncogene homolog (NRAS) oncogenes in the cell lines (Almeida et al., 2020). These results show that EBV may contribute to the progression of TC through the modulation of the expression of oncogenes. The EBV infection also alters the immune microenvironment of PTC by increasing its titer in tumor-promoting immune cells, like M2 macrophages and regulatory T cells (Xie et al., 2020). These results suggest that EBV infection may lead to enhanced immune escape of PTC by altering the immune microenvironment of PTC.

### 1.2 Human herpesvirus-6A

The HHV-6 is a widespread virus with two strains, Human herpesvirus-6A (HHV-6A) and HHV-6B, which exhibit differential cellular tropism and receptor (Caselli and Di Luca, 2007; Jasirwan et al., 2014). Although initially considered lymphotropic, HHV-6 has also been detected in endothelial cells, macrophages, salivary glands, and the brain (Caselli and Di Luca, 2007), with unique biological characteristics and disease associations (Ablashi et al., 2014). For instance, HHV-6B infects more than 70% of children by the age of three (Zerr et al., 2005), while HHV-6A with unclear prevalence is acquired later in life and has a potential link to Alzheimer’s disease and unexplained infertility (Marci et al., 2016; Readhead et al., 2018). Additionally, HHV-6A uses the enveloped gH/gL/gQ1/gQ2 glycoprotein complex to bind to the cellular CD46 receptor, unlike HHV-6B, which rarely binds to CD46 (Jasirwan et al., 2014). However, both strains are possible triggers for various autoimmune diseases, including multiple sclerosis, autoimmune connective tissue diseases and HT (Seyyedi et al., 2019).

**Figure 1:**
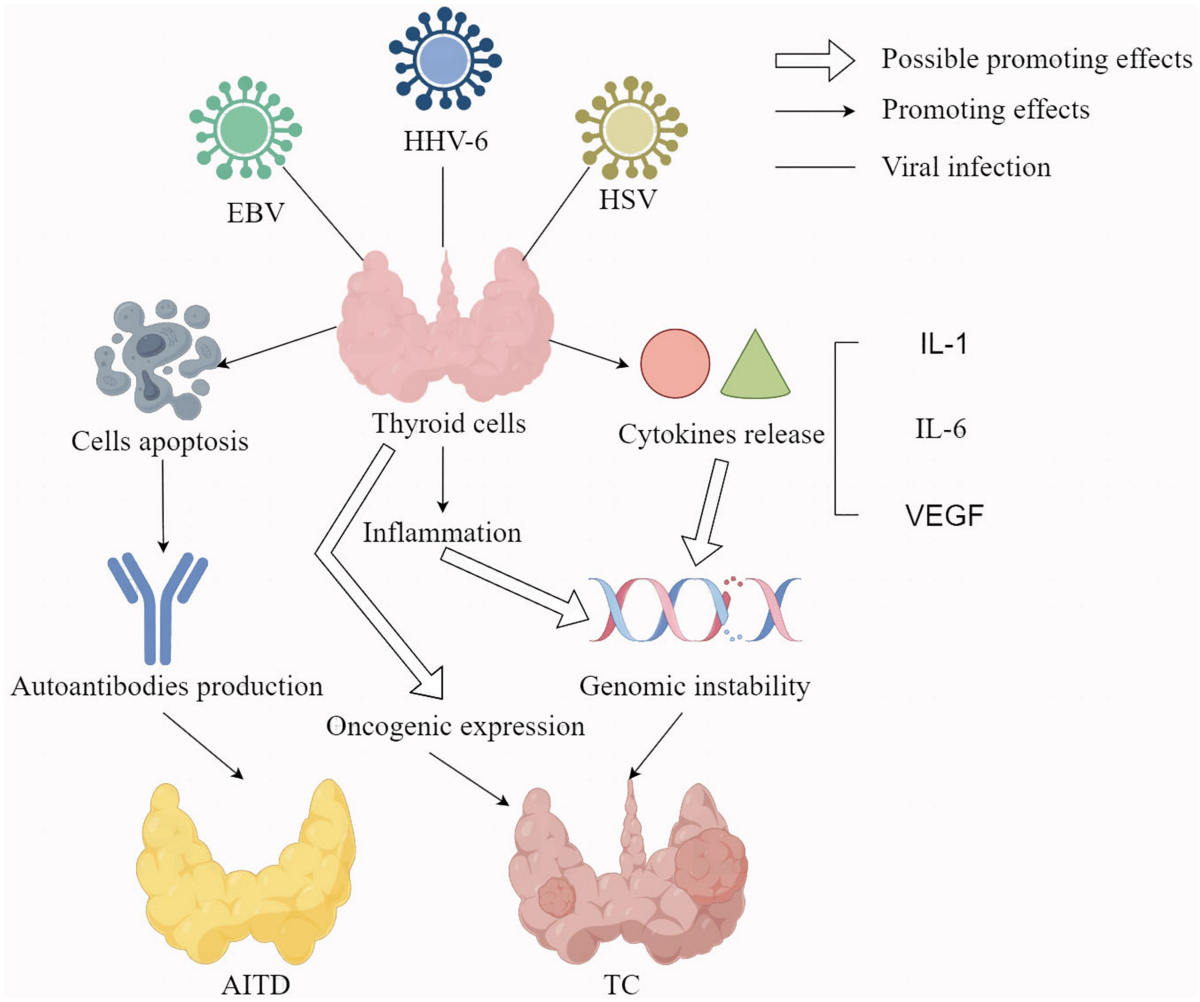
The potential mechanisms of EBV, HHV-6, and HSV infection leading to thyroid diseases. Hollow arrows represent possible promoting effects; Solid arrows represent promoting effects; Solid lines represent viral infections; EBV: Epstein–Barr virus; HI-IV-6: Human herpesvirus-6; I-ISV: herpes simplex virus; VEGF: vascular endothelial growth factor; AITD: autoimmune thyroid disease; TC: thyroid cancer.

The HHV-6A also invades the thyroid follicular epithelial cells where it replicates, inducing the expression of human leukocyte antigen (HLA) class II antigens, thereby enhancing natural killer (NK)-mediated cytotoxicity. The NK-mediated killing of thyroid cells infected by HHV-6A is more efficient in HT and is accompanied by an augmented T-cell response against the HHV-6 U94 protein (Caselli et al., 2012). These findings suggest a possible involvement of HHV-6A in the pathogenesis of HT. Although HHV-6A has been detected in certain tumors, indicating a potential link with cancer, its exact contribution to carcinogenesis remains unclear and warrants further exploration. The HHV-6A may contribute to the occurrence and development of TC by establishing inflammatory and immunosuppressive microenvironments of TC cells (Eliassen et al., 2018). This is attributed to the enhanced release of inflammatory cytokines, oncogenic cytokines and/or the miRNAs implicated in the activation of carcinogenesis (Eliassen et al., 2018). HHV-6A infection may also alter the tumor microenvironment (TME) of TC cells by promoting the production of several pro-inflammatory and pro-oncogenic cytokines in TC cells (Mardente et al., 2023). HHV-6A also enhances the secretion of vascular endothelial growth factor (VEGF) and IL-1 beta (Figure 1). VEGF is a cytokine with pro-tumorigenic, pro-angiogenic, and immune-suppressive properties (Apte et al., 2019), while IL-1 beta contributes to carcinogenesis by facilitating angiogenesis, epithelial-mesenchymal transition, and remodeling of the TME into an immunosuppressive state (Zhang and Veeramachaneni, 2022). Indeed, immune dysfunction can impair the ability of the immune system to suppress tumor initiation, while chronic inflammation can facilitate all the carcinogenesis process, including the progression to more aggressive cancer forms (Greten and Grivennikov, 2019). These inflammation molecules can induce genomic instability, thereby increasing the likelihood of loss-of-function mutations in tumor suppressors like TP53 or proteins involved in DNA damage repair signals like breast cancer type 1 susceptibility protein (Mehrotra and Mittra, 2020). These results *in vitro* prove that HHV-6A is likely to be involved in TC progression. However, evidence from cohort surveys or testing of patient samples is still needed.

### 1.3 Alpha human herpesvirus

Alpha human herpesvirus (αHHV) refers to a family of herpes viruses, including varicella zoster virus (VZV), HSV-1, and HSV-2, which infect humans and establish latency within the neurons following the primary infection phase. Between 1999 and 2004, HSV-1 and HSV-2 had 57.7 and 17.0% incidence, respectively, in the United States of America (Ajavon et al., 2015). Although most symptoms of HSV-1 and HSV-2 infections remain subclinical, complications such as encephalitis caused by HSV-1 reactivation account for nearly 10% of all cases (Martínez et al., 2007). The HSV-1 and HSV-2 pathogens cause unsightly and painful oral and genital lesions (Hsia et al., 2011). HSV-1 has been frequently detected in TC cells, which become increasingly susceptible to HSV infection as they progress toward more malignant forms (Jensen et al., 2010). Although the mechanisms underlying HSV reactivation are still not fully elucidated (Goel et al., 2002), decreased thyroid hormone due to emotional and physical stresses has been identified as its potential triggers (Hsia et al., 2011). A possible link between thyroid hormone dysfunction and αHHV reactivation in hospitalized patients has also been suggested (Hsia and Hsia, 2014). Varicella zoster virus is a neurotropic virus, which establishes latency in ganglia’s sensory neurons after primary infection, typically manifesting as varicella during childhood. Decades later, latent VZV may reactivate, causing zoster or shingles and several severe ocular and neurological complications (Laing et al., 2018).

Some studies indicate that αHHV is closely associated with thyroid dysfunction, AITD, and TC (Jensen et al., 2010). HSV DNA has also been detected in thyroid tissues from patients with autoimmune conditions, such as GD and HT (Thomas et al., 2008). In the context of thyroid carcinogenesis, the replication of HSV in epithelial cells is linked to the RAS/mitogen-activated extracellular signal-regulated kinase (MEK)/MAPK signal activation *in vitro* (Smith et al., 2000), a pathway which is frequently activated in TC. The HSV infection also occurs more frequently in PTC or FTC cells than in normal thyroid cells and can activate NF-κB and pAKT signaling like some thyroid oncogene mutations (Figure 1) (Jensen et al., 2010). In addition, the positivity of HSV-2 was correlated with metastases, BRAF mutation and RET/PTC rearrangements in TC. Therefore, αHHV infection is related to TC but does not initiate it.

The main mechanisms of αHHV-induced AITDs include the promotion of nonspecific inflammation and apoptosis. For the promotion of nonspecific inflammation, HSV infections can enhance the efficiency of autoreactive T cell activation and activate the innate immune system by up-regulating the expression of HLA on antigen-presenting cells, promoting the expression of co-stimulatory molecules, and facilitating the migration of dendritic cells from peripheral tissues to secondary lymphoid organs (Thomas et al., 2008). The persistent activation of the innate immune system in response to the herpes viral infection can also exacerbate autoimmune disorders via chronic immune-mediated autoantigen release and tissue damage. Furthermore, HSV infections can lead to apoptosis of thyroid cells, leading to the release of self-antigens and triggering an autoimmune response (Sanfilippo et al., 2004). This apoptotic effect may be related to the expression of tumor necrosis factor receptor-1 on the surface of CD4 + lymphocytes infected with HSV (Thomas et al., 2008).

### 1.4 Human papillomavirus

Human papillomavirus (HPV) comprises diverse small DNA viruses that exhibit tropism for epidermal squamous epithelia, mucosal surfaces, thyroid tissues and tumors (Archin Dialameh et al., 2021). Its infection leads to the development of malignant carcinomas and benign warts, particularly in the upper aerodigestive tract and the anogenital region (Harden and Munger, 2017). More than 200 distinct HPV types have been identified and classified based on their oncogenic potential into high-risk types, which are strongly related to malignant carcinoma development, and low-risk types, which predominantly lead to benign warts (Lu et al., 2020). Women who received the HPV vaccine exhibit a significantly higher risk of developing HT (Hviid et al., 2018), suggesting a correlation between HPV infection and HT, though how HPV induces HT is unclear. The HPV infection may also be involved in the development of TC, though the results of related studies are inconsistent. The HPV DNA was also detected in 13.4% of PTC samples and 3.8% of benign thyroid nodules (Archin Dialameh et al., 2021; Yang et al., 2024).

### 1.5 Hepatitis C virus

HCV infection causes hepatitis C, which globally occurs in 110 million people, with about 80 million people having chronic viral infection (Gower et al., 2014). The HCV infection also represents a significant cause of chronic liver disease, including hepatocellular carcinoma (HCC) and cirrhosis, which causes about 703,800 deaths yearly. This accounts for one-third of the total HCC mortality (GBD 2013 Mortality and Causes of Death Collaborators, 2015). The HCV belongs to the genus Hepacivirus and family Flaviviridae and possesses a short, single-stranded positive-sense RNA genome of 9.6 kb (Rao et al., 2020). Due to its high degree of concealment and low awareness rate, HCV infection often progresses to chronic hepatitis C (CHC), which may subsequently contribute to the pathogenesis of various autoimmune diseases, including autoimmune liver disease (Rowan et al., 1994), AITDs (Obermayer-Straub and Manns, 2001), cryoglobulinemia (Roccatello et al., 2018), and hypothyroidism (Molleston et al., 2013).

Several studies have suggested a link between CHC and thyroid dysfunction, with CHC patients having higher levels of T3 and T4, anti-thyroid peroxidase antibodies, anti-thyroglobulin antibodies, subclinical and overt hypothyroidism (Antonelli et al., 2004; Indolfi et al., 2008; Danilovic et al., 2013). However, not all studies have found a significant association between CHC and thyroid dysfunction, with some studies indicating insignificant differences between the frequency of AITD among CHC patients with the controls but higher levels of total T3 and T4, which correlate with thyroxine-binding globulin (Danilovic et al., 2013). Another study in untreated children with vertically acquired CHC infection found a higher prevalence of subclinical hypothyroidism than controls, but no association with anti-thyroid autoantibodies or autoimmune disease family history (Indolfi et al., 2008).

There is also a significant association between HCV and PTC, with a higher risk observed in females and individuals aged 50 years or older, although the mechanism behind this association is not fully understood (Montella et al., 2003; Antonelli et al., 2007). Further studies also reveal a higher prevalence of TC in HCV patients, particularly in those with thyroid autoimmunity, showing the importance of incorporating thyroid monitoring of these patients. However, available evidence is not sufficient to prove that HCV infection directly causes TC. Nevertheless, HCV infection may lead to AITD due to the treatment with interferon-alpha (IFN-α), and the release of CXCL10, which is triggered by the infection itself. IFN-α plays a significant role in the pathogenesis of AITD by activating various immune cells, including macrophages, lymphocytes, and neutrophils, and enhancing the production of chemokines and cytokines, particularly IL-6, which has been implicated in AITD. Further, IFN-α upregulates the expression of MHC class I, leading to cytotoxic activation of T cells, tissue damage, and inflammation. The ligation of CD40 on thyrocytes further promotes IL-6 secretion and T-cell activation, contributing to thyroid inflammation. Additionally, IFN-α can promote autoimmunity by altering immunoglobulin production and impairing T regulatory cell function. While HCV infection may exacerbate this process by inducing endogenous IFN, further increasing thyroid auto-antibody production and potentially triggering AITD in susceptible individuals, the combined effect of endogenous and exogenous IFN may lead to clinically significant AITD (Jadali, 2013).

The release of CXCL10 is also a potential contributing factor that links HCV infection to the development of AITD. The CXCL10 is a potent chemoattractant for IFN-gamma-secreting Th1 lymphocytes that is produced by hepatocytes in inflamed regions of patients with chronic viral hepatitis, and recruits T cells to liver lesions. CXCL10 may play a key role in the initial phases of AITD since it is expressed in inflammatory cells and thyrocytes of GD patients, with the higher serum levels in HT patients (Jadali, 2013). IFN-gamma stimulates thyroid follicular cells from GD patients to induce the secretion of CXCL10, further supporting its link in AITD pathogenesis (Ferrari et al., 2019). Furthermore, HCV may exhibit partial amino acid sequence homology with thyroid tissue antigens, thereby promoting autoimmunity in HCV-infected individuals (Hsieh et al., 2000). For instance, the amino acid sequences 647–653 of thyroglobulin (No. U93033) and 3,047–3,053 of HCV type 1 (No. D10749), as well as sequences 1,047–1,054 of thyroglobulin and 2,647–2,654 of HCV type 2 (No. D10750), and sequences 471–476 of microsome (No. E15820) and 471–476 of HCV type 3 (No. D00944), exhibit identical structures (Hsieh et al., 2000).

### 1.6 Human immunodeficiency virus

The HIV infection is characterized by decreased CD4 cell count and immune dysfunction, leading to an elevated risk of opportunistic infections and malignancies (Shmakova et al., 2020; Le Hingrat et al., 2021). Globally, 38 million individuals were infected with HIV in 2019, while 690,000 deaths occurred from HIV infection and AIDS-related diseases (Tian et al., 2023). Owing to the utilization of highly active antiretroviral therapy (HAART), HIV infection has evolved into a controllable chronic illness (Mohamed et al., 2025), with an extended life expectancy, making the patients to confront the challenges of comorbidities or concurrent disorders (Ghosn et al., 2018; Pillay et al., 2020).

Thyroid dysfunction and abnormal levels of TH have been described among the diverse endocrine abnormalities in HIV (Danoff, 2006; Unachukwu et al., 2009; Zaid and Greenman, 2019). Many thyroid dysfunctions are also associated with HIV infection, including subclinical or clinical hypothyroidism (Emokpae and Akinnuoye, 2018; Properzi et al., 2019; Dutta and Kalita, 2023), sick euthyroid syndrome, isolated low FT4 levels (Mayer et al., 2007; Parsa and Bhangoo, 2013; Sebastian et al., 2018), subclinical and clinical hyperthyroidism (Wang et al., 2014; Mohamed et al., 2025) and TC (Liu et al., 2023). Additionally, the immune reconstitution may also cause GD (Vos et al., 2006), unlike TC.

The decreased CD4 count may be the main link between some thyroid dysfunction and HIV progression. For example, the mean CD4 cell count was significantly lower in overt and subclinical hypothyroidism than in hyperthyroidism, sick euthyroid syndrome patients and normal people (Gangannavar et al., 2020). In addition, patients with HIV and subnormal FT4 had substantially fewer CD4 counts than those with normal FT4 (Mohamed et al., 2025). These findings suggest an inverse association with the intensity of hypothyroidism symptoms and CD4 count, but the mechanisms need to be further explored.

The HAART therapy, which improves HIV prognosis, also affects the metabolism of thyroid hormone, though its impact on hypothyroidism remains controversial and inconsistent, while high levels of FT3, subclinical hyperthyroidism and GD have been reported (Beltran et al., 2003). More abnormalities due to subclinical hypothyroidism and low levels of FT4 in HAART patients have also been reported (Madeddu et al., 2006). Further studies also support the lack of association of HAART with thyroid disease. For example, only 2.5% of 1,565 HIV patients treated with HAART studied developed overt hypothyroidism (Madge et al., 2007). Akinsete et al. (2019) also showed that the duration of HAART was not associated with thyroid dysfunction. The proportion of male subjects in the former study was relatively high (79%), and the proportion of thyroid dysfunction was not low (24.5%). The rate of thyroid dysfunction in the latter group did not change over time, but the study was limited to children (Akinsete et al., 2019).

### 1.7 Human parvovirus B19 virus

The B19V is a virus belonging to the Parvoviridae family with a non-enveloped, small and single-stranded DNA (Jalali et al., 2022) and a positive rate of IgG antibody, which increases with age (Nekoua et al., 2023). The virus infects nearly 90% of adults over 60 years, with more frequent infection in females than males (Kerr, 2016) and is mainly transmitted via blood transfusion and the respiratory tract. Acute B19A infection has also been identified as a causative agent for erythema infectiosum in children, hydrops fetalis crisis, and erythroblastopenia (Young and Brown, 2004).

The B19V is frequently detected in the thyroid tissue of AITD patients, revealing its potential role in the disease pathogenesis (Weider et al., 2022), while seropositive children with type 1 diabetes mellitus infected with B19V exhibit a significantly higher prevalence of thyroid autoantibodies than controls (Chandramoorthy et al., 2024). The markedly elevated prevalence of the B19V NS1 gene in non-AITD patients relative to healthy controls, along with the increased viral load observed in the thyroid tissue of AITD patients, also implies that B19V infection may contribute to the development of thyroid disorders (Gravelsina et al., 2019). An association between B19V and PTC or ATC has also been demonstrated by the detection of the B19V capsid protein in the cancerous tissues (Adamson et al., 2011).

The mechanisms by which B19V infection triggers autoimmune diseases include molecular mimicry, the presentation of self-antigens produced by B19V-induced apoptosis, and the activation of the VP1 protein (Kerr, 2016). Several studies have reported significant positive correlations between B19V IgG and thyroid autoantibodies, including thyroid peroxidase and thyroglobulin antibodies, in adults with newly diagnosed GD and HT (Heidari and Jami, 2021). An association of B19V infection with HT has also been demonstrated in animal experiments (Mori et al., 2011). Studies show that B19V infection activates and up-regulates the expression of NF-kB and IL-6, while antibodies against B19V capsid antigen can react with cytokeratin in thyroid epithelial cells, thereby damaging the thyroid and causing HT (Wang et al., 2010). These results confirm the association between B19V infection and HT, but do not provide direct evidence of B19V infection and HT pathogenesis. Therefore, further direct evidence is needed to determine whether B19V infection can cause HT.

### 1.8 Severe acute respiratory syndrome coronavirus 2

The coronavirus disease 2019 (COVID-19), caused by Severe acute respiratory syndrome coronavirus 2 (SARS-CoV-2), not only affects the respiratory system, leading to pneumonia, but also impacts many extrapulmonary systems, including the thyroid (Esmaeilzadeh et al., 2022). The first reported symptom of thyroid dysfunction is subacute thyroiditis (SAT), which occurred approximately 2 weeks after a patient was diagnosed with mild, symptomatic SARS-CoV-2 infection (Brancatella et al., 2020). A previous autopsy study involving five patients who succumbed to SARS-CoV infection revealed significant architectural disruptions in the thyroid gland in 2007 (Wei et al., 2007). Given that SARS-CoV-2 is a newly identified β-coronavirus with approximately 79% genomic sequence identity to SARS-CoV (Hu et al., 2021), concerns have been raised regarding its potential to induce thyroid dysfunction. The primary receptors for SARS-CoV-2, angiotensin-converting enzyme 2 (ACE2), along with its co-receptor transmembrane protease serine 2 (TMPRSS2), are widely expressed throughout the body, including some endocrine organs like the thyroid and pituitary glands.

SARS-CoV-2 is considered a potential trigger of SAT (Brancatella et al., 2020), painless thyroiditis (Mondal et al., 2023), GD (Mateu-Salat et al., 2020) and HT (Brancatella et al., 2023). Patients with SAT after SARS-CoV-2 infection have been reported worldwide in over 100 instances, especially those aged 40 and above, with more incidences in females (Brancatella et al., 2020). The painless thyroiditis was first described as ‘SARS-CoV-2-related atypical thyroiditis’, with thyrotoxicosis and low serum TSH concentrations, requiring highly intensive care (Muller et al., 2020). Another study has also reported cases of painless thyroiditis occurring in patients with COVID-19, which differs from the painful thyroiditis in several aspects (Mondal et al., 2023). More than 20 GD cases have been documented globally, typically manifesting 1–2 months post-SARS-CoV-2 infection, although some cases coincided with acute infection of SARS-CoV-2. Together with reports of other autoimmune disorders emerging shortly after the diagnosis of COVID-19, these findings have elicited concerns regarding GD potentially triggered by SARS-CoV-2. In contrast to GD, fewer cases of HT related to COVID-19 have been documented, partly because patients with mild forms of the disease remain undiagnosed and unreported (Brancatella et al., 2023).

The potential molecular mechanism of thyroid dysfunction caused by SARS-CoV-2 is related to its receptor, ACE2. The discovery of SARS-CoV-2 and ACE2 in thyroid follicular cells, including thyroid tissues with histological features of SAT, indicates the potential for SARS-CoV-2 to infiltrate thyroid cells and directly cause thyroid damage (Lui et al., 2024). Although these reports may suggest the potential association between thyroiditis and COVID-19, the lack of concurrent thyroid function data in these studies restricts further interpretation and conclusive findings. Furthermore, ACE2 is also expressed in the hypothalamus and pituitary glands, rendering them vulnerable to direct damage by SARS-CoV-2 and possibly leading to thyroid dysfunction due to reduced levels of TSH. The SARS-CoV-2 genome has also been observed in the hypothalamic glands of deceased COVID-19 patients (Bellastella et al., 2022), unlike in the pituitary glands, where such evidence remains limited (Capatina et al., 2023).

Other studies have revealed other potential molecular mechanisms of COVID-19-induced thyroid dysfunction, like cytokine storm. COVID-19 is closely linked to significant inflammatory responses, commonly referred to as the ‘cytokine storm’, where the immune system triggers an unregulated inflammatory reaction that is harmful to host cells (Croce et al., 2021). Specifically, certain cytokines, such as IL-6 and TNF, may influence the hypothalamic–pituitary–thyroid axis (Soldevila et al., 2022), thereby contributing to non-thyroidal illness syndrome (NTIS). This impacts the HPT axis, leading to the central suppression of TSH secretion, which in turn results in decreased circulating levels of TSH (Scappaticcio et al., 2021).

The development of AITD following SARS-CoV-2 infection may be associated with molecular mimicry (Brancatella et al., 2023) and “antigen spreading” (Clarke et al., 2022). Molecular mimicry occurs in genetically susceptible people, where an immune response directed against SARS-CoV-2 antigens may potentially trigger an autoimmune reaction (Brancatella et al., 2023), due to sequence homology between SARS-CoV-2 and tissue antigens, like thyroid peroxidase (Vojdani et al., 2020). Vojdani et al. (2020) found that SARS-CoV-2 spike protein, nucleoprotein, and membrane protein all cross-reacted with TPO, and many TPO peptide sequences shared homology or similarity with sequences in various SARS-CoV-2 proteins. On the other hand, antigen spreading may occur after SARS-CoV-2-induced thyroid damage, potentially through the expression of ACE2 in the thyroid (Rotondi et al., 2021). This latter phenomenon could also be triggered by the cytokine storm (Meftah et al., 2023). For example, the levels of IL-6, an inflammatory cytokine implicated in the cytokine storm, are similarly elevated in patients with GD (Clarke et al., 2022). These pieces of evidence suggest that SARS-CoV-2 infection plays an important role in the occurrence of thyroid dysfunction.

## 2 Discussion

This review shows evidence of viral infection causing thyroid dysfunction, including AITD, through direct or indirect mechanisms. Whether a virus directly damages thyroid cells often depends on whether the cells express their receptors, like SARS-CoV-2 (Clarke et al., 2022). Other viral infections cause AITD mainly through indirect methods, while some viruses, like EBV, HSV and B19V, induce apoptosis of thyroid cells to produce self-antigens or promote TRAb secretion, which can cause abnormal immune function, leading to the occurrence of AITD in Figure 1 (Sanfilippo et al., 2004; Kerr, 2016; Nagata et al., 2023). Some viruses, like HCV and SARS-CoV-2, may damage thyroid function and cause the occurrence of AITD by affecting the reconstitution of the immune system and mimicking thyroid components (Hsieh et al., 2000; Clarke et al., 2022). Other viral infections cause thyroid dysfunction or AITD by activating the innate immune system, thereby promoting inflammation of cytokine and chemokine release (Horwitz and Sarvetnick, 1999; Jadali, 2013). The release of different inflammatory factors can also cause different consequences. For example, uncontrolled release of inflammatory factors can lead to cytokine storm and produce large amounts of IL-6 and TNF, which directly affect thyroid function by acting on the hypothalamic–pituitary-thyroid axis (Soldevila et al., 2022). Further, the release of inflammatory cytokines activates some TC-related signaling pathways, thereby promoting the development of TC (Jensen et al., 2010).

Among the hypothesized mechanisms of TC caused by viral infection, many oncogene mutations, cytokines and signaling pathways are involved. Viral infections are thought to trigger TC by promoting the expression of some oncogenes, including BRAF, RAS, and TP53. In fact, BRAF mutation-like and RAS mutation-like are the main gene expression features of PTC and FTC, while TP53 mutation is also closely related to ATC (Volante et al., 2021). In addition, viral infection promotes the release of some inflammatory cytokines or oncogenic cytokines, such as IL-1, IL-6 and VEGF. These cytokines not only have the characteristics of proto-oncogenes, but also can promote angiogenesis, EMT transition, and even remodel TME to play an immunosuppressive role. Finally, the mutated oncogenes and released cytokines activated inflammatory and cancer signaling pathways, such as NF-kB, pAKT and RAS/MEK/MAPK signaling pathways. The activation of these signaling pathways promotes the release of inflammatory molecules, impairs immune function, increases genomic instability, and leads to more cancer gene mutations. The potential underlying mechanism by which viral infection is linked to TC is illustrated in Figure 1.

Though HCV infection can cause some thyroid damage, such as nonautoimmune subclinical hypothyroidism, the association between some viral infections and thyroid dysfunction is still controversial. For example, the frequency of thyroid disorders was not different between HCV-infected patients and the control population (Danilovic et al., 2013). However, the incidence of autoimmune thyroiditis was high in the controls, which may have confounded the results, unlike in children, which was similar to the controls (Indolfi et al., 2008).

The association between viral infection and TC is very complex. For example, HHV-6A promotes TC by enhancing the release of inflammatory cytokines, cancer cytokines and miRNAs, while impairing the ability of the immune system to inhibit the occurrence of cancer (Eliassen et al., 2018). Some viruses, such as HHV-6A, HSV and EBV, can not only induce AITD, but also are closely related to the occurrence of TC. Moreover, viral infection may be involved in the anaplastic transformation process of PTC to ATC. For instance, ATC may arise *de novo* or develop from the well-differentiated TC following the accumulation of genetic alterations, through anaplastic transformation (Kondo et al., 2006). Both ATC and PTC share some common driver mutations, like BRAF and RAS (Volante et al., 2021). In addition, ATC has some unique genetic changes, such as TERT promoter and TP53 mutations (Volante et al., 2021). Other studies suggest that PTC cells with some common ATC gene mutations, such as the TERT promoter mutation, are more susceptible to anaplastic transformation (Odate et al., 2021).

Some viral infections, such as HHV-6A, promote anaplastic transformation of PTC into more malignant FTC or ATC (Mardente et al., 2023) due to the up-regulation of miR-146a-5p, miR-155 and miR-222 in HHV-6A-infected PTC cells. In addition, other αHHV infections activate MAPK signaling pathway (Jensen et al., 2010) or cause genomic instability, which can cause PTC cells to exhibit some ATC-specific genetic changes, such as TERT activation (Liu et al., 2018) and TP53 mutation (Mardente et al., 2023). However, studies on the mechanism of viral infection in the anaplastic transformation of TC are still very limited.

The viral infection has been implicated in thyroid dysfunction and the development of AITD and TC, especially anaplastic transformation, which was accompanied by dramatic changes in clinical mortality (ATC). Therefore, identification of specific viral infections that promote anaplasia can help early screen potential ATC patients and carry out antiviral intervention to reduce the conversion of PTC patients to ATC patients. In addition, chronic viral infections such as the hepatitis C virus, which may be an independent risk factor for thyroid disease, should be emphasized. A systematic review involving 3,603 subjects has revealed that anti-thyroid antibody and hypothyroidism prevalence tended to be higher in HCV-infected subjects (Shen et al., 2016). Therefore, it is necessary to screen thyroid antibodies and function regularly in patients with chronic HCV infection. Consequently, understanding the relationship between viral infections and AITD is crucial for improving antiviral therapies and mitigating their potential side effects. Specifically, in cases of HCV infection, the IFN-α treatment for chronic HCV infection may not only exacerbate but also induce latent thyroid disorders. This may increase the incidence of AITD from 20–40% and thyroid dysfunction from 11–15%, respectively (Pastore et al., 2016). The novel approach involving α-IFN-free combination therapy with direct-acting antiviral agents for chronic HCV infection is helpful to improve patient compliance while reducing the risk of AITD development.
